# Supplementary material for: HIVprotI: an integrated web based platform for prediction and design of HIV proteins inhibitors
Source: J Cheminform. 2018 Mar 9;10:12. doi: 10.1186/s13321-018-0266-y (PMC5845081; doi:10.1186/s13321-018-0266-y)
Supplement: Supplementary file 2 — Additional file 2. Source code of HIVProtI web server. [file 13321_2018_266_MOESM2_ESM.zip › HIVprotI_Source-code/hivprotI/weka-3-6-6/doc/weka/core/PropertyPath.Path.html]

PropertyPath.Path


---


|  |  |  |
| --- | --- | --- |
| **Overview** \| **Package** \| **Class** \| **Tree** \| **Deprecated** \| **Index** \| **Help** \| **Weka's home** | |  |
| **PREV CLASS**   **NEXT CLASS** | **FRAMES**    **NO FRAMES**     **All Classes** |  |
| SUMMARY: NESTED \| FIELD \| CONSTR \| METHOD | DETAIL: FIELD \| CONSTR \| METHOD |  |


---


## weka.core Class PropertyPath.Path

```
java.lang.Object
  weka.core.PropertyPath.Path
```

**All Implemented Interfaces:**: RevisionHandler

**Enclosing class:**: PropertyPath

---

``` public static class PropertyPath.Path extends java.lang.Object implements RevisionHandler ```

Contains a (property) path structure

**Version:**
:   $Revision: 4742 $

**Author:**
:   fracpete (fracpete at waikato dot ac dot nz)

---

**Constructor Summary**

`PropertyPath.Path(java.lang.String path)`
  
          uses the given dot-path

`PropertyPath.Path(java.lang.String[] elements)`
  
          uses the given array as elements for the path

`PropertyPath.Path(java.util.Vector elements)`
  
          uses the vector with PathElement objects to initialize with


| **Method Summary** | |
| --- | --- |
| `PropertyPath.PathElement` | `get(int index)`             returns the element at the given index |
| `java.lang.String` | `getRevision()`             Returns the revision string. |
| `static PropertyPath.Path` | `parsePath(java.lang.String path)`             returns a path object based on the given path string |
| `int` | `size()`             returns the number of path elements of this structure |
| `PropertyPath.Path` | `subpath(int startIndex)`             returns a subpath of the current structure, starting with the specified element index up to the end |
| `PropertyPath.Path` | `subpath(int startIndex, int endIndex)`             returns a subpath of the current structure, starting with the specified element index up. |
| `java.lang.String` | `toString()`             returns the structure again as a dot-path |

**Methods inherited from class java.lang.Object**

`equals, getClass, hashCode, notify, notifyAll, wait, wait, wait`

**Constructor Detail**

### PropertyPath.Path

```
public PropertyPath.Path(java.lang.String path)
```

:   uses the given dot-path

    **Parameters:**: `path` - path in dot-notation

---


### PropertyPath.Path

```
public PropertyPath.Path(java.util.Vector elements)
```

:   uses the vector with PathElement objects to initialize with

    **Parameters:**: `elements` - the PathElements to use

---


### PropertyPath.Path

```
public PropertyPath.Path(java.lang.String[] elements)
```

:   uses the given array as elements for the path

    **Parameters:**: `elements` - the path elements to use


**Method Detail**

### get

```
public PropertyPath.PathElement get(int index)
```

:   returns the element at the given index

    :   **Parameters:**: `index` - the index of the element to return **Returns:**: the specified element

---


### size

```
public int size()
```

:   returns the number of path elements of this structure

    :   **Returns:**: the number of path elements

---


### parsePath

```
public static PropertyPath.Path parsePath(java.lang.String path)
```

:   returns a path object based on the given path string

    :   **Parameters:**: `path` - path to work on **Returns:**: the path structure

---


### subpath

```
public PropertyPath.Path subpath(int startIndex)
```

:   returns a subpath of the current structure, starting with the specified
    element index up to the end

    :   **Parameters:**: `startIndex` - the first element of the subpath **Returns:**: the new subpath

---


### subpath

```
public PropertyPath.Path subpath(int startIndex,
                                 int endIndex)
```

:   returns a subpath of the current structure, starting with the specified
    element index up. The endIndex specifies the element that is not part
    of the new subpath. In other words, the new path contains the elements
    from "startIndex" up to "(endIndex-1)".

    :   **Parameters:**: `startIndex` - the first element of the subpath: `endIndex` - the element that is after the last added element **Returns:**: the new subpath

---


### toString

```
public java.lang.String toString()
```

:   returns the structure again as a dot-path

    :   **Overrides:**: `toString` in class `java.lang.Object`
    :   **Returns:**: the path structure as dot-path

---


### getRevision

```
public java.lang.String getRevision()
```

:   Returns the revision string.

    :   **Specified by:**: `getRevision` in interface `RevisionHandler`
    :   **Returns:**: the revision


---


|  |  |  |
| --- | --- | --- |
| **Overview** \| **Package** \| **Class** \| **Tree** \| **Deprecated** \| **Index** \| **Help** \| **Weka's home** | |  |
| **PREV CLASS**   **NEXT CLASS** | **FRAMES**    **NO FRAMES**     **All Classes** |  |
| SUMMARY: NESTED \| FIELD \| CONSTR \| METHOD | DETAIL: FIELD \| CONSTR \| METHOD |  |


---
